# MicroRNA expression patterns unveil differential expression of conserved miRNAs and target genes against abiotic stress in safflower

**DOI:** 10.1371/journal.pone.0228850

**Published:** 2020-02-18

**Authors:** Farshid Kouhi, Karim Sorkheh, Sezai Ercisli

**Affiliations:** 1 Department of Agronomy and Plant Breeding, Faculty of Agriculture, Shahid Chamran University of Ahvaz, Ahvaz, Iran; 2 Department of Horticulture, Agricultural Faculty, Ataturk University, Erzurum, Turkey; Birla Institute of Technology & Science Pilani, K. K. Birla Goa Campus, INDIA

## Abstract

Environmental stresses influence the growth and development of plants by influencing patterns of gene expression. Different regulators control gene expression, including transcription factors (TFs) and microRNAs. MicroRNAs (miRNAs: ~21 nucleotides long) are encoded by miRNA genes transcribed by RNA polymerase II (RNP-II) and play key roles in plant development and physiology. There is little knowledge currently available on miRNAs and their function in response to environmental stresses in safflower. To obtain more information on safflower miRNAs, we initially used a comparative genomics approach and succeeded in identifying 126 miRNAs belonging to 29 conserved families, along with their target genes. In this study, we investigated the expression profiles of seven conserved miRNAs related to drought, salinity, heat, and Cd stress in the leaf and root organs using qRT-PCR, for the first time. Gene Ontology (GO) analysis found that target genes of miRNAs are often TFs such as AP2/ERF and HD-ZIP as well as NAC domain-containing proteins. Expression analyses confirmed that miRNAs can play a vital role in keeping safflower stress-tolerant. Differential expression of miR156, miR162, miR164, miR166, miR172, miR398, and miR408 regulate the expression of their respective target genes. These genes activate several pathways leading to physiological and biochemical responses to abiotic stresses. Some conserved miRNAs were regulated by abiotic stresses. Our finding provides valuable information to understand miRNAs in relation to different abiotic stresses in safflower.

## Introduction

Safflower (*Carthamus tinctorius* L.) is one of the most desirable oilseed crops with remarkable yields (about 32–40% seed oil) [1], and can tolerate environmental stresses, including high temperatures, salinity, and dehydration. This oilseed crop has the ability to grow in most regions of the world, particularlywhere climatic and soil limitations prevent the cultivation of conventional food and cash crops [2, 3]. However, there are only a few published reports on the response of safflower to heavy metal stress. Some of these studies indicate that it may be planted as a hyper-accumulator crop to expedite the amelioration of soils pollution caused by heavy metals such as Cd [4]. Therefore, an understanding of the basis of the drought, salinity, heat, and Cd stress responses in safflower is important, because it can offer insights in to the tolerance mechanisms against these environmental stresses at the molecular level. Considering the discovery of microRNAs (miRNAs) and the recognition of their function in recent years, it is feasible that these may play an important role in safflower development and plant feedback to biotic and abiotic stresses.

MiRNAs include novel ncRNA molecules and are about 21–25 nt in length. They negatively adjust the expression of a wide range of genes at the post-transcriptional level via inhibition of gene translation or repression of target mRNAs through pairing with their target mRNAs[5, 6].Following the report of involvement of miRNAs in the molecular response to some abiotic stresses [7], extensive studies have been conducted, which show that the expression levels of miRNAs have been altered in plants exposed to various environmental stresses such as viral and fungal infections [8, 9], phosphate and nitrogen starvation [10, 11], mechanical stresses [12], drought [13, 14], temperature [15, 16], salinity[17, 18], and heavy metal stress [19, 20]. In addition, researchers have found that plant miRNAs are involved in different physiological activities, for example, flowering time, development of root, and seed germination [21–23]. Gutierrez *et al*.[22] reported that miRNA160 and miRNA167 are genes targeted by the ARF transcription factor through interference with auxin regulation. This showed an important aspect of growth and development of plant under abiotic stress. The expression of AGO, which is controlled by miRNA403, has an antiviral role [24–26]. These are also examples of differential expression of miRNA and gene targets against various environmental conditions [26].

Currently, 463,906 contigs of safflower obtained as findings of safflower Whole Genome Shotgun (WGS) project have been deposited in the GenBank WGS database (based on data collected on April 17th, 2016). Based on valuable WGS resources, using bioinformatics methods, Kouhi *et al*. [27] have identified and reported 107 miRNAs in safflower. Nevertheless, their expression patterns under various environmental conditions have not been exhaustively surveyed.

In the study ahead, Kouhi *et al*.[27] first compared all safflower WGS sequences with plant miRNAs in the mirBase database to identify potential miRNA homologs in safflower. In addition, we identified the potential target genes of miRNA and their potential functions [27]. To confirm the anticipated miRNAs and the mutual relationship between miRNAs and their target genes, as well as analyze the miRNAs expression pattern, SL RT-PCR and qRT-PCR was performed to illustrated the expression levels of seven putative miRNAs and seven target genes, respectively in the root and leaf organs of safflower seedlings under various abiotic stresses (**Table 1**). Recent reports have indicated that the seven mentioned miRNAs (miR156, miR162, miR164, miR166, miR172, miR398, and miR408) play crucial roles in plant responses to both biotic and abiotic stresses [28].

**Table 1:** Listof stress-responsive potential miRNAs and their candidate target genes in safflower.

miRNA	miRNA sequence	Predicted targets
mir156	TGACAGAAGAGAGTGAGCAC	Squamosa promoter-binding(SPL)
mir162	TCGATAAACCTCTGCATCCAG	uncharacterized protein (unknown)
mir164	TGGAGAAGCAGGGTACGTGCA	NAC domain-containing protein (NAC)
mir166	TCGGACCAGGCTTCATTCCCC	HD-ZIP protein REV (HD-ZIP)
mir172	AGAATCTTGATGATGCTGCAT	AP2/ERF domain-containing protein (AP2)
mir398	TGTGTTCTCAGGTCGCCCCTG	Superoxide dismutase [Cu-Zn] 1 (CSD1)
mir408	TGCACTGCCTCTTCCCTGGCT	Cupredoxin(CUP)

## Results

### Experimental validation of predicted miRNAs with putative targets and analysis of their expression patterns in response to abiotic stress by qRT-PCR

The qRT-PCR analysis was carried out to verify the sequences of the amplicon obtained by bioinformatic analysis and investigate the expression patterns of seven miRNAs including miR156, miR162, miR164, miR166, miR172, miR398 and miR408 and their target genes at vegetative phases under salt, Cd, heat, and drought stresses.

In the present study, concentrations of Cd, Na, and K, expression of the HSP70 protein and changes in leaf relative water content (RWC)were used to measure the effectiveness of stressors on the various physio-molecular characteristics of plantlets.RWC is a criterion of stress-adaptation that describes osmoregulation in response to water stress in higher plants. During drought treatment, plantlets showed a significant decrease in RWC after 12, 24 and 48 h of readily available water uptake(Table 2).Following heat shock at 42°C, HSP70 expression increased in leaf and root organs(**S1 Table**).Flame spectrometryanalysisindicates that Na and Cd accumulated significantly in the root than in leaves, especially under more severe stress (Table 3).In addition to these results, the results obtained on proline content show that plantlets were remarkably affected by the stresses and the intensity of stresses had forced them to respond (**S2 Table**).Therefore, the change patterns, measured by these physiological and molecular parameters prove that the difference seen in qPCR results below was affected by the applied stressors.

**Table 2:** Effect of different drought levels on relative water content safflower. Two-tailed t-test was used to compare the three drought treatments with the (100% FC) control sample, in leave.

Treatment levels	RWC
Control	85.49%
12h	66.33%**
24h	55.18%**
48h	39.83%**

**showed significant at 1%

**Table 3:** Salt tolerance measure (mg/g dw) and cadmium concentration (mg/kg dw) in leave and roots of safflower.

	Concentration	Organ	
		Root	Leaf
**Na**^**+**^	Control	1.063267	0.9525
	75mM	5.0968**	3.4104**
	150mM	9.3261**	3.81985**
**k**^**+**^	Control	26.77033**	44.06427
	75mM	23.48433*	34.00643**
	150mM	18.8029**	31.79607**
**k**^**+**^**/Na**^**+**^	Control	25.1774	46.2616
	75mM	4.6076**	9.9713**
	150mM	2.0161**	8.3239**
**Cadmium**	Control	1.15	0.9
	5mg/L	52.8**	3.96**
	20mg/L	103.5**	5.27**

**, and * showed significant at 1%, 5%, respectively.

### qRT-PCR analysis of candidate drought-responsive miRNAs and their targets

**Fig 1:**
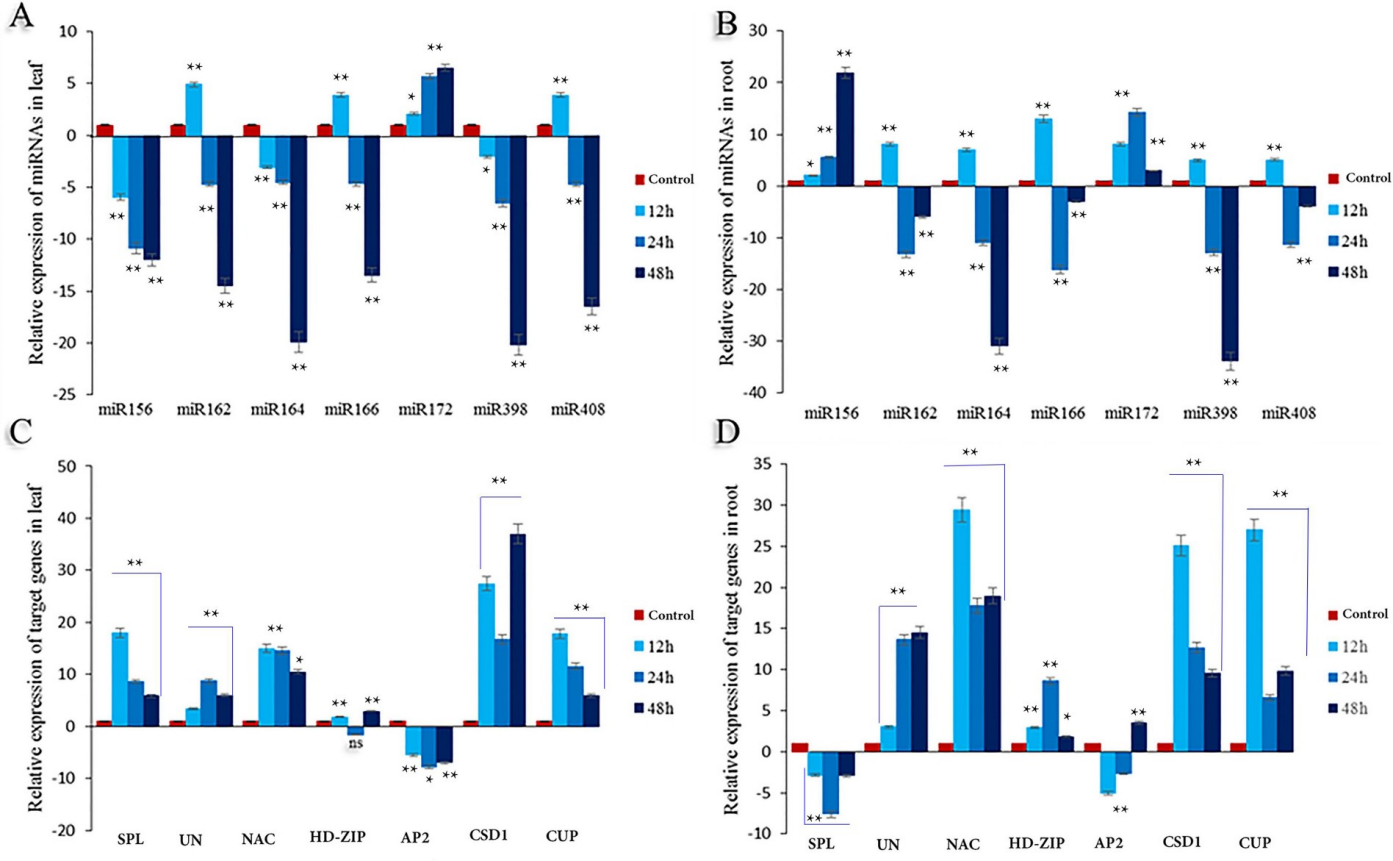
The relative expression level of selected seven miRNAs and their targets in the leaf and root of safflower under drought stress. For drought stress, Plantlets were subjected to water deficit for 0 (control), 12, 24 and 48 hours after readily available water (RAW) uptake. (**A**) Differential expression of miRNAs in the leaf. (**B**) Differential expression of miRNAs in the root. (**C**) Differential expression of the target gene in the leaf. (**D**) Differential expression of target genes in the root. All fold changes are marked with **, * and ns showed significant at 1%, 5% and non-significant, respectively.

Quantification of seven candidate mature miRNAs and their targets was performed for root and leaf organs using three biological replicates for each stress level. All seven miRNAs showed differential expression at each drought stress level and were significantly down-regulated(*P*<0.05)in leaves (except the expression of miR172,miR162,miR166, andmiR408 after 12h)with the lowest expression levels seen at 48 h. In roots exposed to drought, miRNAs show increased expression at 12h and then were down-regulated as the duration of dehydration continued. The expression patterns of miRNAs (except miR156),were almost identical in both organs. Generally, most of the up and down regulation of miRNAs resulted in changes in the order 22 to 34 fold (**Fig 1**).

The expression of target genes has increased in both organs and at all levels of stress (except SPL and AP2).Superoxide dismutase [Cu-Zn] 1 (CSD1) and NAC domain-containing protein (NAC) showed a similar trend in leaf organs. The respective expression of these two genes were up-regulated in response to the first drought shock, although it eventually decreased within the 24hwindow.Similarly,in the root expression of NAC, Cupredoxin (CUP) and CSD1 are up-regulated, though with a downward trend (**Fig 1**).

### qRT-PCR analysis of candidate heat-responsive miRNAs and their targets

Based on the fold change and *P*-value (≤0.05), in leaf organ, the expression level of miRNAs in response to heat shock varies significantly. The expression patterns of miR156, miR164, and miR398 at all levels were up-regulated within a range of 8 to 25-foldin leaf organs. Between miRNAs, only the expressions of miR162 and miR172in both organs were down-regulated. Similarly, in root organs, the expression of miR164 and miR398after the beginning of the treatment, reduced continuously at the 3 and 6 h marks.

**Fig 2:**
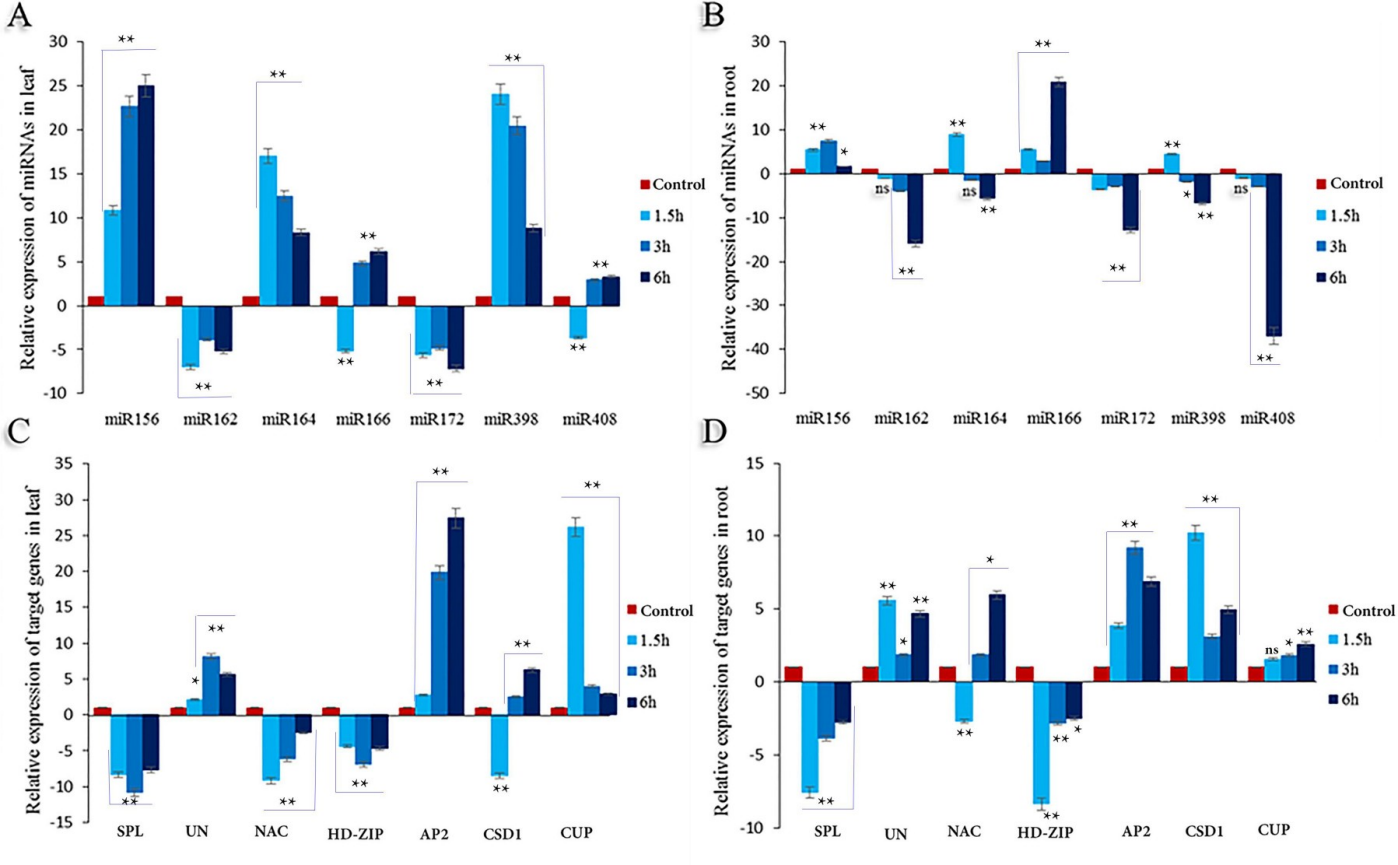
The relative expression level of selected seven miRNAs and their targets in the leaf and root of safflower under heat stress. For heat stress Plantlets subjected to 25± 1°C (control) and 42 ± 1°C for 1.5, 3 and 6 hours. (**A**) Differential expression of miRNAs in the leaf. (**B**) Differential expression of miRNAs in the root. (**C**) Differential expression of target gene in the leaf. (**D**) Differential expression of the target genes in the root. All fold changes are marked with **, * and ns showed significant at 1%, 5% and non-significant, respectively.

The expressions of the targets are often incremental in both organs, especially at the root. Similarly, the expression pattern of HD-Zip and SPL are down-regulated in photosynthetic and non-photosynthetic organs (**Fig 2**).

### qRT-PCR analysis of candidate salt responsive miRNAs and their targets

**Fig 3:**
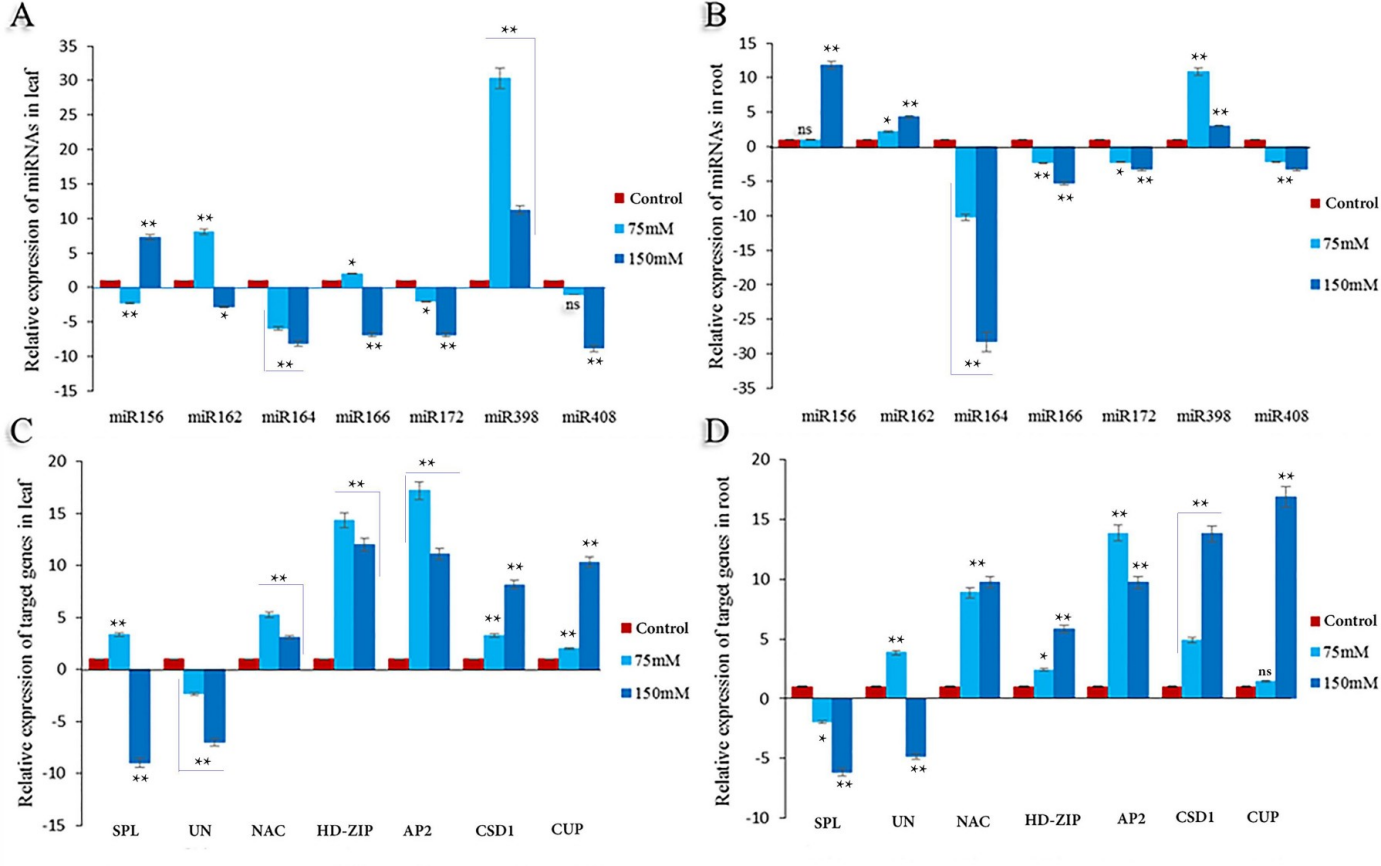
The relative expression level of selected seven miRNAs and their targets in leaf and root of safflower under salt stress. Salt treatments were applied in 0 (control), 75 and 150 mM NaCl concentration (**A**) Differential expression of miRNAs in the leaf. (**B**) Differential expression of miRNAs in the root. (**C**) Differential expression of target gene in the leaf. (**D**) Differential expression of the target genes in the root. All fold changes are marked with **, * and ns showed significant at 1%, 5% and non-significant, respectively.

Salinity treatments have led to differential expression of miRNAs and potential gene targets in leaves and roots (**Fig 3**).Two expression change patterns were observed in leaves and roots under salt treatments. The expression of miR164 was down-regulated at 75 mM and 150mM NaCl concentration treatment and vice versa; the salt induced the expression of miR398.Generally, the expression of all miRNAs at the 150mM NaCl concentration reached the lowest value.

The expression of miRNA targets in leaves was induced under the treatment, although they showed a downward trend at the 150mM NaCl concentration level. However, unlike other targets, uncharacterized proteins (unknownproteins) showedlower levels in the leaves. The expressions of an unknown protein, along with NAC, AP2, and CSD1 were significantly increased at the lowest NaCl concentrations but declined slightly in the severe saline treatment. Meanwhile, the SPL, HD-ZIP and CUP expression was continually stimulated by increasing the salt concentration.

### qRT-PCR analysis of candidate Cd-responsive miRNAs and their targets

**Fig 4:**
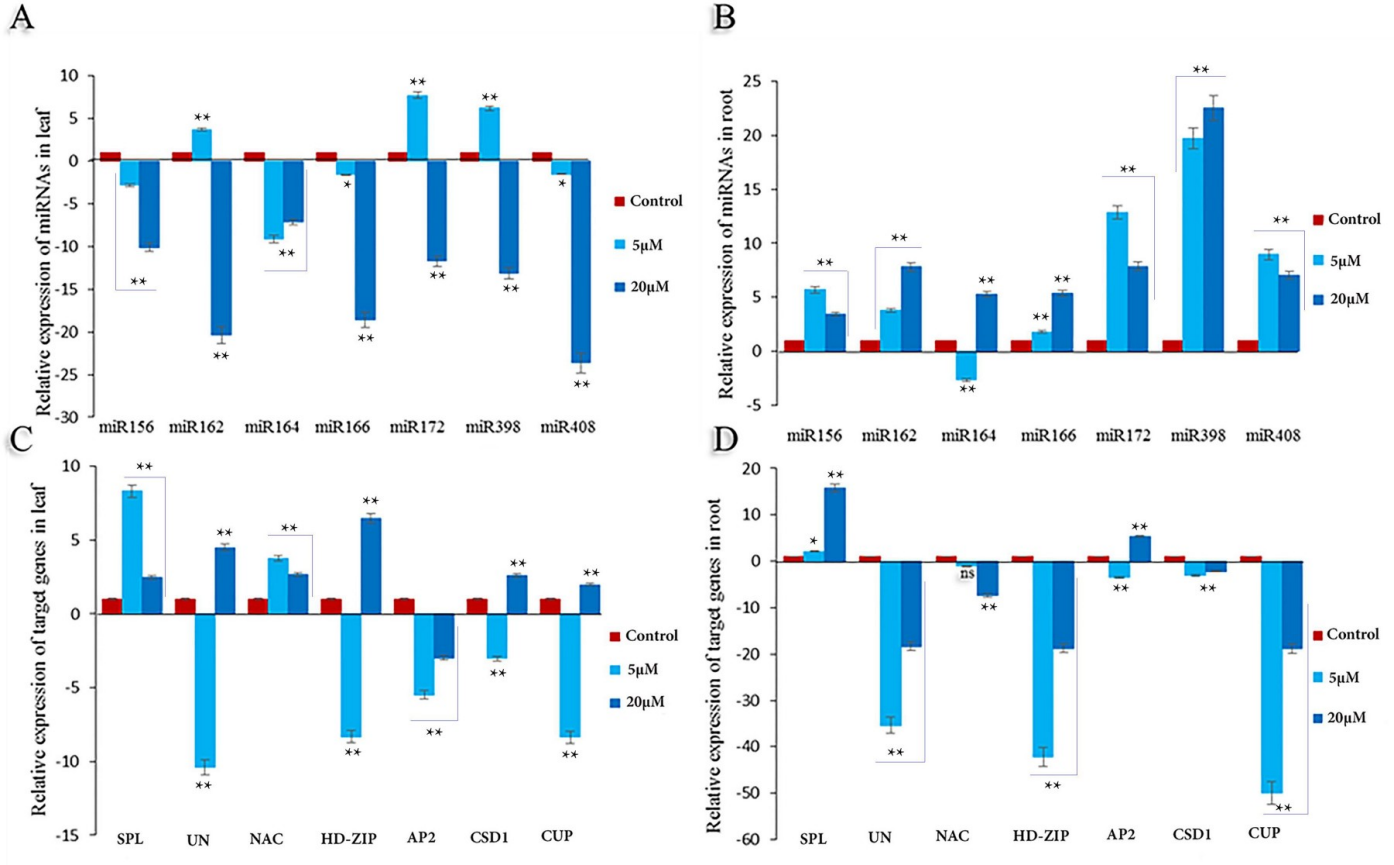
The relative expression level of selected seven miRNAs and their targets in the leaf and root of safflower under cadmium stress. Cadmium treatments were applied in 0 (control), 5 and 20 μM Cd concentration. (**A**) Differential expression of miRNAs in the leaf. (**B**) Differential expression of miRNAs in the root. (**C**) Differential expression of target gene in the leaf. (**D**) Differential expression of the target genes in the root. All fold changes are marked with **, * and ns showed significant at 1%, 5% and non-significant, respectively.

Cd treatment stimulated the expression of miRNAs in the root organ, with documented fold-changes between 1.8 and 26. In leaf tissues, the maximum and minimum values came from miR172 in 5 μM and miR408 in 20 μM Cd, respectively. In addition,the expression level of miR156, miR164, miR166, and miR408 decreased steadily and the maximum reduction was seen at a concentration of20μM.Contrarily,in the root, most of the miRNAs are continually up-regulated(**Fig 4**).

**Fig 5:**
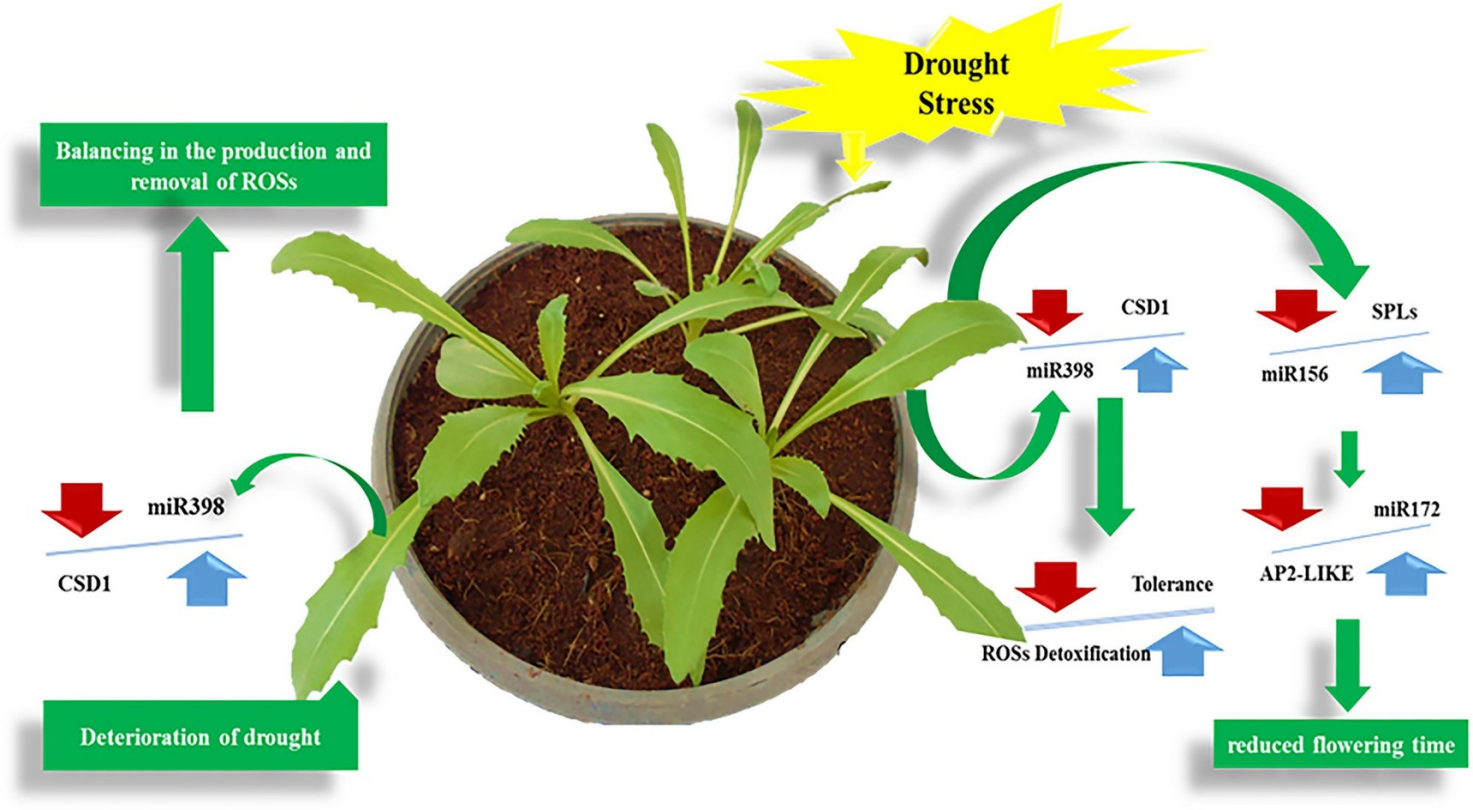
The function of miR398 in removing ROSs arising from drought stress in safflower, and the effect of drought stress on reproductive phase timing in safflower.

Transcripts of the uncharacterized protein, HD-ZIP, and CUP showed a similar trend in root organs. The expression of each of these is reduced at all treatment levels at a rate of less than 18 fold. In addition, Cd suppressed the expression of NAC and CSD1 less intensely (**Fig 5**).

### Heat-map based clustering approach of miRNAs and target genes

**Fig 6:**
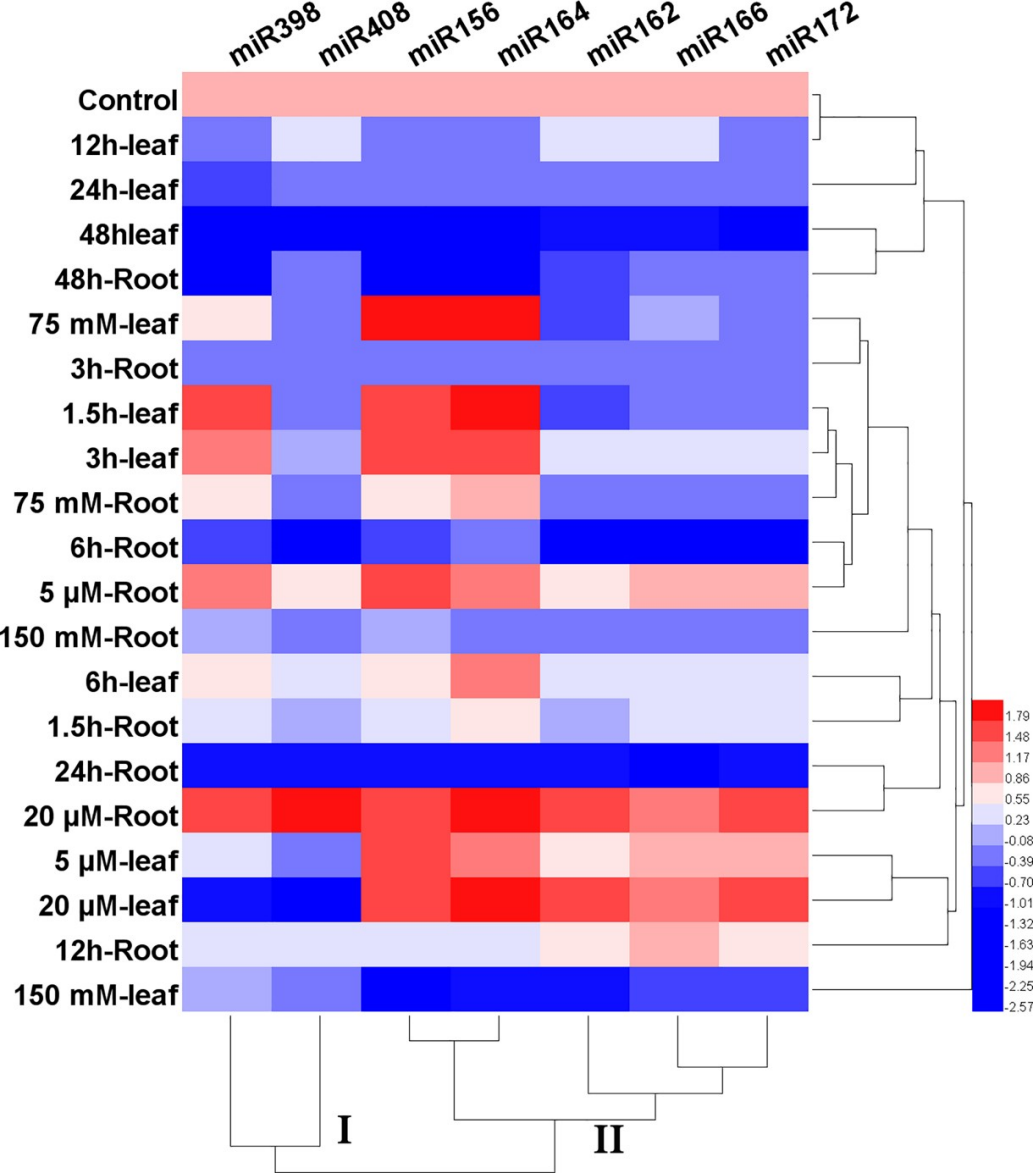
Heat-map based clustering approach of miRNAs. Heat-map based clustering of miRNA expression in different conditions of abiotic stress. The sample and treatment are given in the column and reach row illustrated miRNA.

Heat-map based clustering approach was performed separately for miRNAs and target genes. Specific clusters has to be identified which are stress-responsive in safflower. Expression profile of seven miRNAs in both leaf and root was conducted to explore their expression of tissue-specific profiles to different abiotic stress conditions. Clustering expression patterns of the studied miRNAs showed 2 groups. Cluster I consisted miRNA398 and miRNA408. Cluster II included miRNA166, miRNA172, miRNA162, miRNA164 and miRNA156 and discriminated an alternative pattern in leaves and roots in different conditions stress (**Fig 6**).

**Fig 7:**
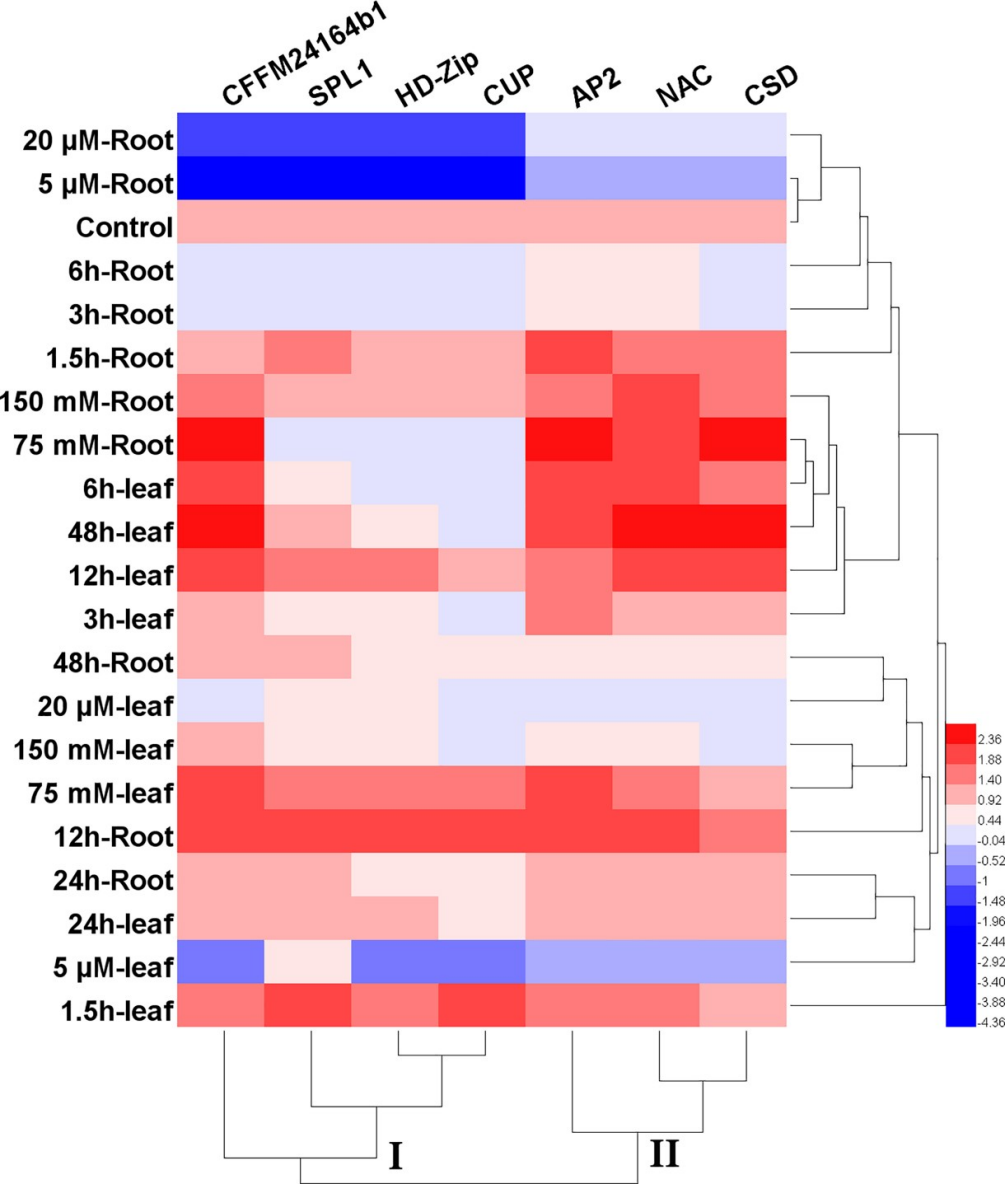
Heat-map based clustering approach of target genes. Heat-map based clustering of target genes expression in different conditions of abiotic stress. The sample and treatment are given in the column and reach row illustrated target genes of miRNAs.

These miRNAs also separately categorized for candidate target genes based on expression patterns in 2 clusters. Cluster I consisted candidate target genes of SPL1, HD-Zip, CUP which have similar patterns in condition stress, but CFFM24164b1 (as unknown protein) was showed different expression pattern. Cluster II visualized AP2, NAC and CSD target genes (**Fig 7**).

## Discussion

The most detrimental factors such as environmental stresses influence the crop production and quality of plant species due to their wide distribution [29]. Crop species due to no-escape from unfavorable stress conditions sounding them, have to develop various physio-molecular mechanisms to cope with stress [30]. Laterally, miRNA have proved new players in tolerance of plant to abiotic stresses such as drought, saline, cold, heat [31] and much attempt has been specified to clarified the role of miRNA in the responses of various abiotic stresses in several plant species [32–35].The status of in RWC, Na^+^, K^+^ and Cd status in both tissues of leaf and root provide additional information on environmental stress conditions in safflower.

In this study, we employed WGS analysis to identify 126potential conserved miRNAs belonging to 29different families in safflower [27]. Of the miRNAs, miR393, miR477, miR530, miR6111, miR6113, and miR6114 have not been previously reported in safflower. In contrast, at least one member of the 23 other miRNA families has already been identified in safflower [36, 37], which has been verified based on the utility of the computational prediction of potential miRNAs.

In later years, a large number of conserved and species-specific miRNAs involved in stress responses have been recognized in many important crops. However, the miRNA expression profiles in safflower organs still had not been investigated in response to abiotic stresses. During this investigation, qRT-PCR indicated that seven miRNAs have different expression models in both leaves and root of plants under stress and normal conditions. This is in accordance to prior results reported in wheat and rice [38, 39]. In fact, miRNAs are expressed only in some organs and in response to environmental stresses [40, 41].

In the present study, expression patterns of the seven miRNAs have been consistent with reports of some previous studies and are inconsistent with some other reports that may be due to differences in plant species and severity or duration of stresses. For example, in response to drought, the expression of miR164, miR398,and miR408 in safflower exhibited results similar to results of high-throughput sequencing and qRT-PCR in *Medicago truncatula*, Arabidopsis, and rice (down-regulated) [11, 42, 43].miR408 in safflower, radish, rapeseed, and soybean roots responded similarly (up-regulated) to Cd stress [44–46].Results obtained on miR172 and miR166 expression under salinity are consistent with observations in radish (down-regulated) [47], and an increase in the levels of mature miRNA was observed for miR156 and miR166 in response to heat, which is concordant with results observed in wheat and barley [48, 49].

The role of miRNA172 during drought, heat, cold and Cd have suggested by several studies [31, 32]. The different gene expression patterns of miRNA172 observed in saline, heat and drought stresses. miRNA172 regulate AP2 gene family and involved in various process including flowering time [44]. Over-expression of miR172 reduced flowering time significantly [23]. We found that miR172 is especially down-regulated in leaf tissues during salt and heat stress while it is up-regulated under drought stress. Beyond this, changes in flowering time and the expression pattern of miR172 have been shown, AP2 domain-containing TFs to be effective in mediating stress tolerance [50].

The miR398 family has shown a variety the patterns of expression in response to stresses in safflower. Interestingly, we found that the level of miR398 was down-regulated after drought treatment in both root and leaf organs, and also has a negative correlation with their target gene (CSD1) indicating a response of tissue-specific directed by this miRNA. Considering that the ‘Sina’ cultivar is a dry land variety, it can be concluded that in this variety, at the molecular level, miR398 can play an important role in drought resistance regulatory networks (**Fig 5**).This is in accordance with previous studies [33].The miR398 family in plants, which is related to detoxification processes, can directly interact with stress regulatory networks because their targets are genes encode Cu/Zn-SOD enzymes (CSD1 and CSD2). In this study, CSD1 was evaluated as a potential target for miR398.A secondary effect caused by most abiotic stresses in plants is the rapid accumulation of reactive oxygen species (ROS) [51]. Superoxide dismutases (SODs) are enzymes that can change the highly toxic superoxide radicals into H_2_O_2_, which is less toxic [52]. Differential expression of miRNA398 in response to environmental stresses conditions have been shown in different plant species including *Brassica*, Arabidopsis, *Nicotina*, Wheat and sunflower [27, 32–35]. All of these results are deduced that miRNA398 may be involved in detoxification of ROS accumulation during abiotic stresses.

The miR408 has been described in different plant species to be differential expressed in responses to different abiotic stresses. In this study, miRNA408 expression was measured under drought, saline, heat and Cd stresses [43, 36]. Contrary to our previous conception, our results demonstrated an increased expression of miR408 in response to salinity and drought. It is possible that another similar condition is required for drought tolerance and salinity.On the other hand, the expression of miR408 in the root organs after Cd treatment is up-regulated.

The miR408 family was suggested as Cu-miRNA [37]. In Arabidopsis at low and high Cu concentration, Cu-miRNA accumulated and disappeared, respectively [38, 39].The main target genes of miR408 encode blue copper proteins, including those in the phycocyanin family [53, 54]. The biological function of Cu is entirely related to the reconstruction of its properties. Since free copper is toxic, even in small amounts, its homeostasis is controlled by precise molecular mechanisms. Copper ion (II) is reduced to copper (I) before entering the cell through high-affinity copper transporters of the CTR family [55]. In Cupredoxin, Cu^+^ is stabilized by a constrained His2Cys coordination environment [56]. Up-regulation of miR408 due to abiotic stress decreased the expression of genes associated with unnecessary cuproproteins (such as plantacyanin, cupredoxin, uclacyanin, and LAC3); this process leads to enhanced copper level available to those cuproproteins that are essential for coping with stress, such as the Cu/Zn SODs [41]. Jovanovic *et al*. [57] was reported miRNA408 expression down-regulated in pea upon drought stress. The results of this study provide sufficient evidence of an important role for miR408 in response to abiotic stress.

In this study, miRNA164 show expression down-regulated under drought and salinity stresses. This discovery is similar to down-regulation miRNA164 in *M*. *truncatula* after drought treatment [58]. In our study, NAC TF is one of the targets identified for miR164 in safflower. The results of qRT-PCR show a negative correlation between miR164 and the NAC gene in response to salinity and drought stress. Fang *et al*. [58] found that miR164 and NAC TFs show a significant function in regulating drought tolerance in rice, such that up-regulation of miR164 against NAC TFs can cause drought sensitivity. Further studies have shown that NAC TFs directly bind to the promoters of drought-responsive genes [59, 60]. Also, transgenic plants that expressed miRNA164-resistant NAC1 in Arabidopsis increased the number of lateral roots [61]. Therefore, it is conceivable that miR164 expression suppression in safflower may assist in increasing the root/shoot ratio under salt and drought stress.

The miRNA165 and miRNA166 family members regulate the expression class III homeodomain-leucine zipper (HD-ZIP III) TF which are regulate leaf morphology and auxiliary meristem initiation [62, 63–65]. In this study, miRNA166 was down-regulated in response to salinity which is in agreement with results obtained in maize [66].n former studies, miR166 displayed temporal up-regulation in leaves and roots in response to heat stress. Expression of miR166 showed an inverse correlation with its target HD-ZIP protein, REV. The down-regulation of miR166 in salt-tolerant safflower and the up-regulation of the HD-ZIP transcript might enhance salt tolerance.

Interestingly, the pattern of miR162 was different in each stress treatment. Its expression was up-regulated in salinity. In contract, miR162 illustrated down-regulated under heat stress. This is proffer that miR162 may specified to stress-specific response explored by this miRNA. Meanwhile, the mRNA encoding DCL1 has been known as the target of miR162 [67], but this result was not seen in safflower. In this study, the expression of an unknown gene is considered the potential target of miR162. It was observed that miR162 expression under salt stress, like maize [66], is up-regulated. Also, the unknown protein expression changes in response to abiotic stressors significantly, which indicates its involvement in the stress response. On the contrary, the negative correlation between miR162 and the unknown protein confirms the existence of a relationship between them and can be better understood.

Several studies have suggested role for miR156 during environmental stress conditions in various plant species [68–71]. In this study, the target of miR156 was SPL TF which may alter expression of downstream genes. Our findings suggest that the expression of miR156 has decreased in particular in the leaves of plantlets under drought stress. Perhaps it can be concluded that if the safflower seedlings are subjected to mild drought stress for a long time, they can continue to survive with an increased flowering rate. In contrast to drought stress, heat stress has led to an increase in mir156 expression. We observed down-regulation of SPL genes in leaves and roots under salinity and heat stress indicating that miR156 accelerates the cleaveage of SPL o modulate stress responses in safflower. In Arabidopsis, miR156 temporally controls phase change and trichome development by targeting the distribution of SPL transcription factors [72, 73]. Both miR156 and miR172 were shown to participate in the regulation of developmental timing in Arabidopsis [74]. Since the transcription of miR172 was directly regulated by SPLs transcription factors [74], increasing its expression leads to the expression of miR172, which in turn targets and cleaves AP2-LIKE transcripts. By decreasing the expression of the SPL TFs, the expression of the miR172 was also reduced. The findings showed that high temperature can change the expression of all components in the miR156-SPL-miR172-AP2 pathway through a complex mechanism and probably delay the flowering of young safflower seedlings. We propose that miR156 and miR172 expressions in apical meristems should also be studied to validate these results.

The identification of miRNA in safflower under different environmental stress conditions in the previous study by Kohi *et al*. [27] and expression analysis in the present study, would be a starting point to understand regulatory mechanisms and biological functions of identified miRNA in the future.

## Methods

### Plant materials and biological samples

Safflower seeds (*Carthamus tinctorius* L., ‘Sina’ (PI- 537598)), obtained from the Deputy of Kermanshah, Sararood Dry Land Agricultural Research Institute, Iran) were surface-sterilized for 10 min with sodium hypochlorite (5%), then completely washed with distilled water and in order to obtain uniformity, the seedlings were germinated at room temperature and under dark conditions for 3 days in sterile Petri dishes on water-moistened filter papers. After germination, the seedlings were cultured in different media.

To apply the heat stress treatment, three sprouted seeds were planted in jardinières filled with sterile and moist cocopeat and for the drought treatment three germinated seed were planted in pots containing 1kg of sandy clay loam soil. Safflower seedlings were grown under normal conditions in the greenhouse at Shahid Chamran University in Ahvaz, Iran. Irrigation was performed according to seedlings’ water requirements in cocopeat and soil, using ¼ strength Hoagland’s solution [75] and water, respectively. To prevent the accumulation of nutrient elements and occurrence of high salinity in cocopeat, irrigated using a solution containing water and Hoagland’s solution in 1:4 ratio.

For salinity and Cd stress, after germination, seedlings were transferred to 5Lplastic containers containing¼ strength Hoagland’s nutrient medium and grown hydroponically for six weeks. To avoid contamination and decreases in nutrient concentrations, the nutrient solution was changed twice a week. pH of the solution was maintained at ~5.8.

### Abiotic stress treatments

Drought, heat, saline and heavy metals are the main stress sources in the plant kingdom [76, 77]. The safflower plantlets (8–10 leaf stages) were exposed to the above-mentioned stress treatments.

### Drought stress

To create drought stress, plantlets were exposed to a water deficit through water restriction over 0 (100% FC as a control group), 12, 24, and 48 h after readily available water (RAW) uptake (**S1 Fig**), and then recovery was conducted by re-irrigation. To characterize the status of plant water, RWC was determined in the youngest fully expanded leaf, according to Catsky [78].

### Heat stress

Heat stress was applied by transferring watered plantlets to a growth chamber with 70% relative humidity. The plantlets were subjected to 25± 1°C (control group) and42 ± 1°C (heat stress) for 1.5, 3, and 6 h (**S1 Fig**). To confirm the effect of heat stress on roots and leaves, plant tissue was analyzed to detect the presence of Hsp70 protein-coding gene (GenBank ID: EL400852.1).Heat shock proteins (HSPs) are a category of conserved and ubiquitous proteins and their expression arises in response to abiotic stress like high temperature [79].

### Saline stress

To impose salt stress, six-week-old plantlets were transferred to fresh culture solution containing 0 mM (control group), 75 or 150 mM of NaCl and the roots and leaves were separately collected from samples after 24 h. In order to investigate different salinity stress levels, 0.1 g of sample from each of the oven-dried leaf and root sample(70°C for 48 h), was digested with 10 mL 0.1N glacial acetic acid; then, Na^+^ and K^+^ content of samples (mg g^-1^dw) were measured by flame photometer (Jenway, PFP-7, Cole-Parmer Ltd, Stone, Staffordshire, UK) [80].

### Heavy metal stress

Cd stress treatments were performed under the same conditions by feeding hydroponic solution supplemented with 0 (control), 5 or20 μM CdSO_4_. After 24h of Cd treatment, leaves and roots of treated plants were harvested separately. To assess the severity of stress, combusted samples (550°C for 2/5 h) were digested with 2N hydrochloric acid. Cd content was measured as a measure of stress by flame atomic absorbance spectrometry (Perkin-Elmer Spectrophotometer, Model 460, USA).

### Determination of proline content

The content of proline from 500 mg of leaf and root samples was extracted separately using 3% (w/v) sulphosalicylic acid (SA) and then estimated with the application of ninhydrin reagent following the procedure demonstrated previously [81]. The proline absorbance was measured at 520 nm. The proline concentration was ascertained by comparison with a standard curve and expressed as mg proline g^−1^fw.

### Total RNA extraction

Total RNA was extracted from the fresh leaf and root samples using a total RNA extraction kit (EZ Spin Column Total RNA Isolation Kit, BioBasic Inc., Canada), according to the manufacturer’s instructions, and then treated with DNase I (Takara) to eliminate remaining gDNA. The quality of the RNA samples was appraised by electrophoretic separation on a 0.8% agarose gel. The RNA concentration was ascertained using a biophotometer spectrophotometer (Eppendorf, Germany).

### Stem-loop reverse transcription (SL RT) and quantitative Real-Time PCR

The expression patterns of 7 mature miRNAs in response to abiotic stress were considered by SL RT-PCR.SL RT-PCR primers for *C*. *tinctorius* mature miRNAs (miR156, miR162, miR164, miR166, miR172, miR398, and miR408) were designed manually according to the procedure illustrated by Chen *et al*. [82]) and Varkonyi-Gasic *et al*. [81](**S3 Table**).Immediately after the total RNA extraction, the miRNA-specific SL RT reactions were performed pursuant to Varkonyi-Gasic *et al*. [83] via minor adjustments, using PrimerScript^TM^RT reagent kit (TAKARA, Japan).The RT reaction was performed as 16°C, 30 min for 1 cycle; 30°C, 30 s; 42°C, 30 s; 50°C, 1 s for 60 cycles and terminated by incubation at 85°C for 5 min.

The qRT-PCR was used with SYBR *Premix Ex Taq*^TM^ (TAKARA, Japan) and monitored with the Master Cycler System (ABI, Biosystem, USA). Using 1 μL RT stem-loop cDNA products, quantitative PCR reactions were performed as 5 μL SYBR *Premix Ex Taq*^TM^ (2×), 0.2 μL forward (10 μM), 0.2 μL reverse (10 μM) primers, 0.2 μL Reference Dye II (50X) and 3.4 μL nuclease-free water were mixed. Forward primers were specifically designed for each individual miRNA and the reverse primer was universal for all sets of stem-loop primers [83]. The qRT-PCR reactions were done based on the following conditions; 5 min at 95°C, 40 cycles for 5 s at 95°C for denaturation, 10 s at 60°C for annealing and 5 s at 72°C for elongation. The melting curve analysis was performed by denaturation at 95°C for 15 s, followed by default increasing ramp rate of the instrument from 60°C to 95°C.All of these reactions were performed for three biological repeats with technical duplicates. Relative expression levels for each sample were obtained using the comparative Ct (2^−ΔΔCt^) method, also β-actin and glyceraldehyde-3-phosphate hydrogenase (GAPDH) were used as two reference genes to normalize expression values [84]. The statistical analysis was performed using SPSS 22 software at a 5% level of significance. The heat map of gene expression of miRNAs and target gens was illustrated using HemI 1.3.7 [85].

### Expression analysis of miRNA targets genes by qRT-PCR

To distinguish the expression of miRNA targets and the possibility of discovering new miRNA target genes under salinity, Cd, heat and drought stress in safflower, the expression levels of the miRNA-associated predicted sequences were assayed with qRT-PCR. Primers were designed for target and reference genes using Primer3, in accordance with the criteria described by Thornton and Basu [84] (**S4 Table**).

Total cDNAs synthesis was carried out with 1μg RNA using PrimerScript^TM^RT reagent kit (TAKARA, Japan) according to the manufacturer’s instructions. The qRT-PCR was performed using gene-specific primers in a total volume of 10 μL as follows: 5 μLSYBR *PremixExTaq*^™^ (Takara, Japan), 0.2 μL Reference Dye II (50X), 0.2 μL of each specific primers, 1 μL of the cDNAs as a template. Reaction conditions were as follows: 95°C for 5 min, 40 cycles at 95°C for 10 s, 55°C for 30 s, and 72°C for 30 s, followed by a final 10 min extension at 72°C and then was done disassociation stage (melting curve analysis).

## Supporting information

S1 Table Expression pattern of the HSP70-related protein in safflower after heat stress. (DOCX)

S2 Table Effect of different stress on levels of free proline in the leave and roots of safflower (mg/g fw). (DOCX)

S3 Table Primers used for SL RT–PCR. (DOCX)

S4 Table Primers designed for targets and reference genes. (DOCX)

S1 Fig Seedlings 6 weeks of safflower affected by drought and heat stress: **Drought: A**. normal–first level: 12h- second level: 24h- third level: 48h; **Heat: B**. normal–first level: 1.5h- second level: 3h- third level: 6h.
(DOCX)

## Abbreviations

TFs: Transcription factors
miRNAs: MicroRNAs: ~21 nucleotides long
RNP-II: RNA polymerase II
GO: Gene Ontology
WGS: Whole Genome Shotgun
RWC: Relative water content
CSD1: Superoxide dismutase [Cu-Zn] 1
NAC: NAC domain-containing protein
CUP: Cupredoxin
ROS: Reactive oxygen species
SODs: Superoxide dismutases
HD-ZIP: Homeodomain-leucine zipper
RAW: Readily available water
SA: Sulphosalicylic acid
GAPDH: glyceraldehyde-3-phosphate hydrogenase
SL RT: Stem-loop reverse transcription
SL RT-PCR: Stem-loop reverse transcription PCR
