# Supplementary material for: MicroRNA expression patterns unveil differential expression of conserved miRNAs and target genes against abiotic stress in safflower
Source: PLoS One. 2020 Feb 18;15(2):e0228850. doi: 10.1371/journal.pone.0228850 (PMC7028267; doi:10.1371/journal.pone.0228850)
Supplement: S1 Table — (DOCX) [file pone.0228850.s001.docx]

**S1 Table: Expression pattern of the HSP70-related protein in safflower after heat stress. For apply heat stress, The Plantlets were subjected to 42 ± 1◦C (heat stress) for 1.5, 3 and 6 hours. To confirm the effect of heat stress on root and leaf organs of safflower was used of Hsp70 protein-coding gene (GeneBank ID: EL400852.1).**

| **Treatment levels** | **relative expression in leaf** | **relative expression in root** |
| --- | --- | --- |
| Ctrl | 1 | 1 |
| 1.5h | 8.63116465** | 43.97109952** |
| 3h | 2.62984693** | 11.35389591** |
| 6h | 5.06064117** | 15.69195711** |

**, showed significant at 1%.
