# Supplementary material for: MicroRNA expression patterns unveil differential expression of conserved miRNAs and target genes against abiotic stress in safflower
Source: PLoS One. 2020 Feb 18;15(2):e0228850. doi: 10.1371/journal.pone.0228850 (PMC7028267; doi:10.1371/journal.pone.0228850)
Supplement: S2 Table — (DOCX) [file pone.0228850.s002.docx]

**S2 Table: (A, B, C, D) | Effect of different stress on levels of free proline in the leave and roots of safflower (mg/g fw).**

**A**

| **Treatment levels** | **heat stress** | |
| --- | --- | --- |
|  | **Root** | **Leaf** |
| ctrl | 29.8 | 34.45 |
| 1.5h | 28.39 | 35.79 |
| 3h | 31.12 | 37.1* |
| 6h | 30.41 | 38.82* |

**B**

| **Treatment levels** | **drought stress** | |
| --- | --- | --- |
|  | **Root** | **Leaf** |
| ctrl | 29.8 | 34.45 |
| 12h | 39.84** | 89.87** |
| 24h | 47.1** | 102.51** |
| 48h | 85.59** | 159.6** |

**C**

| **Treatment levels** | **cadmium stress** | |
| --- | --- | --- |
|  | **Root** | **Leaf** |
| ctrl | 29.8 | 34.45 |
| 5mg/L | 43.01** | 41.15** |
| 20mg/L | 34.64* | 36.68* |

| **Treatment levels** | **salt stress** | |
| --- | --- | --- |
|  | **Root** | **Leaf** |
| ctrl | 29.8 | 34.45 |
| 75mM | 35.75* | 36.31* |
| 150mM | 60.3** | 60.11** |

**D**

**, and * showed significant at 1%, 5%, respectively.
