# Supplementary material for: MicroRNA expression patterns unveil differential expression of conserved miRNAs and target genes against abiotic stress in safflower
Source: PLoS One. 2020 Feb 18;15(2):e0228850. doi: 10.1371/journal.pone.0228850 (PMC7028267; doi:10.1371/journal.pone.0228850)
Supplement: S3 Table — (DOCX) [file pone.0228850.s003.docx]

**S3 Table: Primers used for SL RT–PCR**

|  |  | **Sequence (5'->3')** | **Length** | **Tm** | **GC%** |
| --- | --- | --- | --- | --- | --- |
| mir156 | RT | GTCGTATCCAGTGCAGGGTCCGAGGTATTCGCACTGGATACGACGTGCTC |  |  |  |
|  | Forward | TGGCGTGACAGAAGAGAGT | 19 | 62.8 | 52.6 |
|  | miR | TGACAGAAGAGAGTGAGCAC |  |  |  |
| mir162 | RT | GTCGTATCCAGTGCAGGGTCCGAGGTATTCGCACTGGATACGACCTGGAT |  |  |  |
|  | Forward | GCCGTCGATAAACCTCTGC | 19 | 62.5 | 57.9 |
|  | miR | TCGATAAACCTCTGCATCCAG |  |  |  |
| mir164 | RT | GTCGTATCCAGTGCAGGGTCCGAGGTATTCGCACTGGATACGACTGCACG |  |  |  |
|  | Forward | TGGTGGAGAAGCAGGGTA | 18 | 62.1 | 55.6 |
|  | miR | TGGAGAAGCAGGGTACGTGCA |  |  |  |
| mir166 | RT | GTCGTATCCAGTGCAGGGTCCGAGGTATTCGCACTGGATACGACGGGGAA |  |  |  |
|  | Forward | CTGTCGGACCAGGCTTCA | 18 | 63.4 | 61.1 |
|  | miR | TCGGACCAGGCTTCATTCCCC |  |  |  |
| mir172 | RT | GTCGTATCCAGTGCAGGGTCCGAGGTATTCGCACTGGATACGACATGCAG |  |  |  |
|  | Forward | ACGGCGAGAATCTTGATGATG | 21 | 62.7 | 47.6 |
|  | miR | AGAATCTTGATGATGCTGCAT |  |  |  |
| mir398 | RT | GTCGTATCCAGTGCAGGGTCCGAGGTATTCGCACTGGATACGACCAGGGG |  |  |  |
|  | Forward | GCAGTGTGTTCTCAGGTCG | 18 | 62.3 | 57.9 |
|  | miR | TGTGTTCTCAGGTCGCCCCTG |  |  |  |
| mir408 | RT | GTCGTATCCAGTGCAGGGTCCGAGGTATTCGCACTGGATACGACAGCCAG |  |  |  |
|  | Forward | CGTTGCACTGCCTCTTCC | 18 | 62.8 | 61.1 |
|  | miR | TGCACTGCCTCTTCCCTGGCT |  |  |  |
|  | Reverse | GTGCAGGGTCCGAGGT | 16 | 63.1 | 68.8 |
