# Supplementary material for: MicroRNA expression patterns unveil differential expression of conserved miRNAs and target genes against abiotic stress in safflower
Source: PLoS One. 2020 Feb 18;15(2):e0228850. doi: 10.1371/journal.pone.0228850 (PMC7028267; doi:10.1371/journal.pone.0228850)
Supplement: S4 Table — (DOCX) [file pone.0228850.s004.docx]

**S4 Table: Primers designed for targets and reference genes**

|  | **Target_Acc** | **primer** | **Sequence (5'->3')** | **Length** | **Tm** | **GC%** | **amplicon L** |
| --- | --- | --- | --- | --- | --- | --- | --- |
| cti-SPL | EL398105.1 | Forward | CAAGTTGGTGGGTTGAGTTATT | 22 | 61.6 | 35.1 | 121 |
|  |  | Reverse | GCATCAGGCTCTGTTTGATTAC | 22 | 61.5 | 45.5 |  |
| cti-unknow | EL386787.1 | Forward | GACACAGAAAGACGACGAC | 19 | 59.9 | 52.63 | 111 |
|  |  | Reverse | CCACCAAGAGCAGCAAAC | 18 | 61 | 55.56 |  |
| cti-NAC | EL374434.1 | Forward | CGGTGGTGTCGTTCTTGG | 18 | 62.4 | 61.11 | 119 |
|  |  | Reverse | TTGGATTGGAGAAGCAGGG | 19 | 61.7 | 52.63 |  |
| cti-HD-ZIP | EL390889.1 | Forward | GCCTATGCTCCAACCACAC | 19 | 62.6 | 57.89 | 94 |
|  |  | Reverse | GCTCGCAAACCACAAGAC | 18 | 61.2 | 55.56 |  |
| cit-AP2 | EL403681.1 | Forward | TATGGCGAACAGTGGAGG | 18 | 61 | 55.56 | 102 |
|  |  | Reverse | GTTTTTGGGTCATTCCGTCC | 20 | 61.4 | 50 |  |
| cti-CSD1 | EL410611.1 | Forward | GTCACAGTAGGCGACGATG | 19 | 61.8 | 57.89 | 100 |
|  |  | Reverse | GGACAACTACAGCCCTTCC | 19 | 61.7 | 57.89 |  |
| cti-Cupredoxin | EL406272.1 | Forward | CTTTGCTTGTCCATCATTCG | 20 | 59.5 | 45 | 103 |
|  |  | Reverse | ATTAGAAGGGGTGGTGGTG | 19 | 61.1 | 52.63 |  |
| cti-hsp70 | EL400852.1 | Forward | GTGAAGGCTGAAGACAAGGC | 20 | 59.12 | 55 | 95 |
|  |  | Reverse | ACCATCCGCTCGATCTCTTC | 20 | 59 | 55 |  |
| Cti-Actin | KJ634809.1 | Forward | TGGAATGGAAGCGGCTGGTA | 20 | 62 | 55 | 182 |
|  |  | Reverse | CTTGATCTTCATACTGCTTGG | 21 | 60 | 52 |  |
| Cti-GAPDH | AID52923.1 | Forward | AGTGTTGCCAGCCCTTAATG | 20 | 61 | 50 | 103 |
|  |  | Reverse | AGCCTTCTTCTCAAGCCTTAC | 21 | 61 | 47.2 |  |
