# Supplementary material for: MicroRNA expression patterns unveil differential expression of conserved miRNAs and target genes against abiotic stress in safflower
Source: PLoS One. 2020 Feb 18;15(2):e0228850. doi: 10.1371/journal.pone.0228850 (PMC7028267; doi:10.1371/journal.pone.0228850)
Supplement: S1 Fig — Seedlings 6 weeks of safflower affected by drought and heat stress: Drought: A. normal–first level: 12h- second level: 24h- third level: 48h; Heat: B. normal–first level: 1.5h- second level: 3h- third level: 6h. (DOCX) [file pone.0228850.s005.docx]

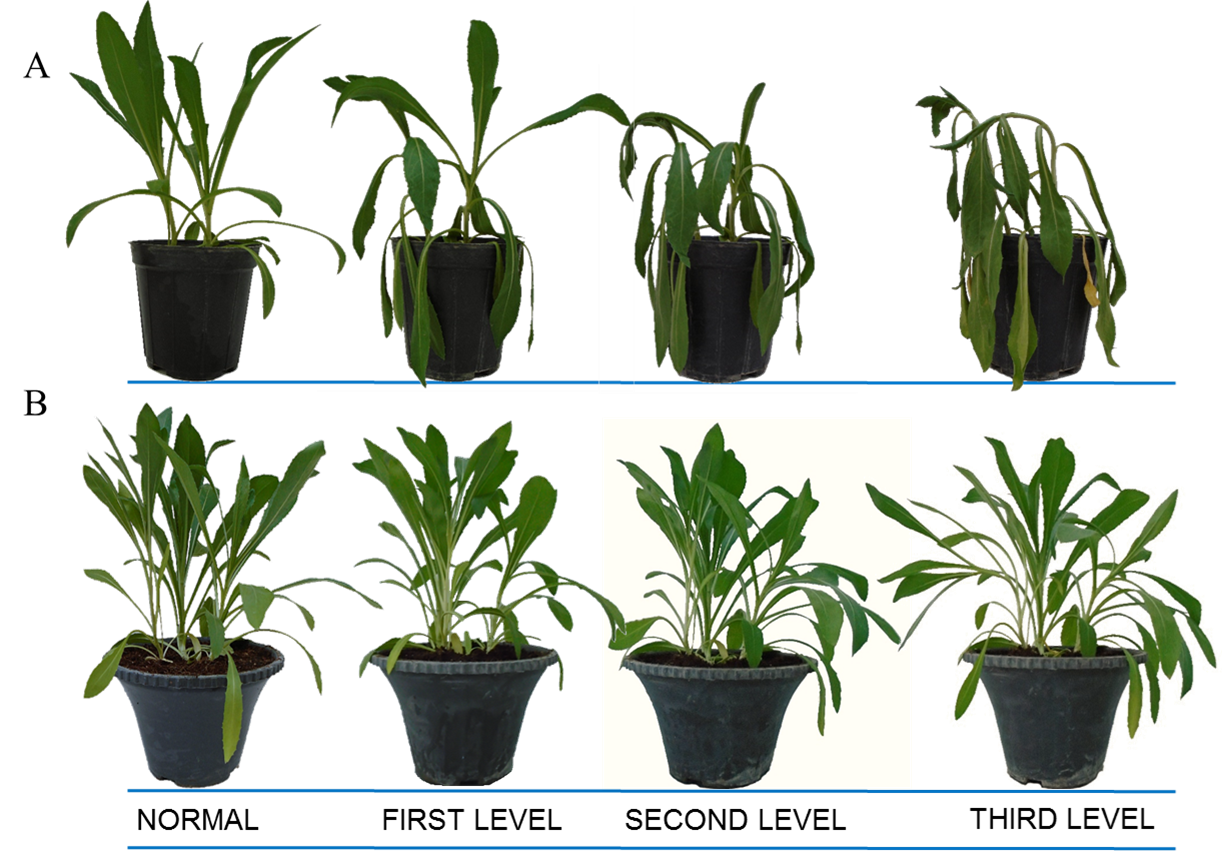


**S1 Fig: Seedlings 6 weeks of safflower affected by drought and heat stress.**

**Drought: A**. normal– first level: 12h- second level: 24h- third level: 48h

**Heat: B**. normal– first level: 1.5h- second level: 3h- third level: 6h
